# Goal-Directed Fluid Therapy and Perioperative Epidural Analgesia in Low-Risk Patients Undergoing Elective Colorectal Surgery: Short-Term Clinical Outcomes From a Retrospective-Prospective Cohort Study

**DOI:** 10.7759/cureus.99819

**Published:** 2025-12-22

**Authors:** Agathi Karakosta, Evangelia Kaminioti, Maria Riga, Panagiotis Briassoulis, Aggeliki Pantazi, Tatiana Sidiropoulou, Petros Tzimas, Georgia G Kostopanagiotou

**Affiliations:** 1 Department of Anesthesiology, University Hospital of Ioannina, Faculty of Medicine, University of Ioannina, Ioannina, GRC; 2 Second Department of Anesthesiology, Attikon University Hospital, National and Kapodistrian University of Athens, Athens, GRC; 3 Department of Anesthesiology, Faculty of Medicine, School of Health Sciences, University of Ioannina, Ioannina, GRC

**Keywords:** colorectal surgery, enhanced recovery, epidural analgesia, goal directed fluid therapy, length of hospital stay, low-risk patients

## Abstract

Enhanced Recovery After Surgery (ERAS) protocols emphasize perioperative fluid management to optimize outcomes following major abdominal surgery. This study aimed to investigate the effect of thoracic epidural analgesia on hospital length of stay in low-risk patients undergoing open elective colorectal surgery managed with goal-directed fluid therapy (GDFT). A retrospective-prospective single-center cohort study was conducted, enrolling low-risk (American Society of Anesthesiologists (ASA) I-II) patients scheduled for open elective colorectal surgery. Forty-nine patients were prospectively assigned to either GDFT with intravenous analgesia (GDFT group) or GDFT with epidural analgesia (GDFT/ED group). Additionally, 72 patient records managed with conventional fluid therapy (CFT), with (CFT/ED group) or without epidural analgesia (CFT group), were retrospectively reviewed. The primary outcome was length of hospital stay; secondary outcomes included intraoperative fluid administration, gastrointestinal recovery, pro-B-type natriuretic peptide (BNP) levels, and in-hospital mortality. Overall analysis showed shorter hospital stay across groups, which did not reach statistical significance (p=0.08), while pairwise comparison demonstrated significantly shorter stay in the GDFT/ED group compared with the CFT group (p=0.048). Gastrointestinal recovery did not differ significantly between groups, although ileus was more frequent in the CFT and CFT/ED groups. Total intraoperative fluid administration was significantly lower in GDFT-managed patients (p=0.006), with no significant difference between the GDFT and GDFT/ED groups. Baseline and postoperative proBNP levels were comparable across groups, and no in-hospital deaths occurred. In conclusion, among low-risk patients undergoing major open elective colorectal surgery, combining GDFT with thoracic epidural analgesia was associated with a trend toward shorter hospital stay. These findings support integrating individualized fluid optimization with effective analgesia within ERAS protocols even for low-risk patients.

## Introduction

Existing evidence highlights the benefits of Enhanced Recovery After Surgery (ERAS) in accelerating recovery, reducing hospital stay, and lowering postoperative morbidity, mortality, and rehospitalization rates [1]. A key component of ERAS is perioperative fluid management, which is essential for gastrointestinal recovery after open colorectal surgery. Both overhydration and underhydration can impair organ function and negatively impact patient outcomes [2].

Goal-directed fluid therapy (GDFT), which relies on dynamic volume assessments, facilitates balanced fluid administration and has been shown to improve perioperative outcomes, particularly in major abdominal surgery [3]. Colonic anastomotic leakage remains a serious complication following colorectal surgery, occurring in up to 20% of cases [4] and significantly increasing morbidity, mortality, length of hospitalization, and healthcare resource utilization. Hypovolemia may lead to tissue ischemia and breakdown at the anastomotic site, while hyperhydration can cause tissue edema, weakening anastomotic integrity. Thoracic epidural analgesia (TEA) may further influence gastrointestinal recovery by modulating splanchnic perfusion through sympathetic blockade. While epidural-induced vasodilation can reduce systemic vascular resistance and arterial pressure, it may also improve microcirculatory blood flow in the splanchnic bed, potentially enhancing tissue oxygenation at the anastomotic site. However, these effects may be offset by compensatory fluid administration or vasopressor use, underscoring the importance of maintaining euvolemia during colorectal surgery. However, most existing research focuses on high-risk patients, with limited data on those at low to moderate risk [4,5].

TEA combined with general anesthesia has been widely used for major abdominal surgery. Given its hemodynamic effects, careful perioperative fluid optimization is particularly relevant when TEA is used. Various clinical and laboratory markers have been used to monitor perioperative fluid status. B-type natriuretic peptide (BNP) levels have been shown to correlate with fluid balance [6] and are associated with morbidity and mortality, as well as hospital length of stay [7,8]. However, BNP represents an indirect marker influenced by multiple factors beyond intravascular volume status, and its utility as a perioperative risk indicator in surgeries involving substantial fluid shifts, such as colorectal procedures, remains inadequately explored.

This study hypothesizes that TEA may contribute to overhydration, as a compensatory response to vasoplegia, potentially impairing gastrointestinal recovery and prolonging hospital stay, a hypothesis that remains controversial in contemporary ERAS-based practice. The primary objective was to evaluate the impact of TEA on hospital length of stay in low-risk patients managed with GDFT. Secondary outcomes included total intraoperative fluid volume administered, proBNP levels as a marker of fluid balance, postoperative adverse events including gastrointestinal dysfunction, and all-cause in-hospital mortality. Additionally, records of patients managed with conventional fluid therapy (CFT), with or without TEA, were retrospectively reviewed for comparison.

## Materials and methods

This is a hybrid prospective-retrospective observational analytical single-center cohort study with quantitative methodology, conducted in patients undergoing elective open colorectal surgery within an ERAS protocol. The study adheres to the Declaration of Helsinki and has been approved by the hospital’s ethics committee (06-15/09/2016). Written informed consent has been obtained from all prospective participants. The study is registered on ClinicalTrials.gov (NCT06810648).

Given the hybrid prospective-retrospective design, particular attention was paid to minimizing potential sources of confounding. All study groups were drawn from the same institution and surgical department, within a consistent ERAS framework, and shared identical inclusion and exclusion criteria. To reduce temporal and practice-related variability, retrospective cases were selected from a period immediately preceding the prospective arm and involved comparable surgical procedures and perioperative care pathways. Although fluid administration in the retrospective CFT groups was not protocolized, baseline patient characteristics, surgical complexity, and perioperative management were carefully reviewed to ensure comparability across groups.

Inclusion and exclusion criteria

**Figure 1:**
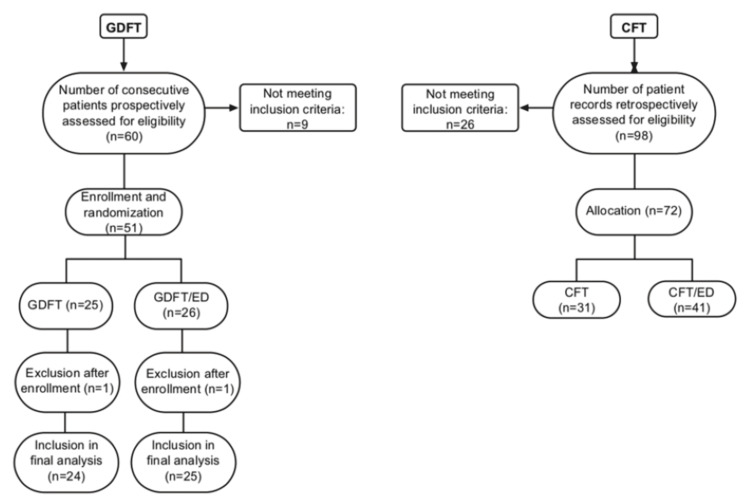
Flow chart of patient selection and recruitment. The diagram summarizes the selection and allocation of patients in both the prospective and retrospective arms of the study. GDFT: goal-directed fluid therapy, CFT: conventional fluid therapy, GDFT/ED: GDFT with thoracic epidural analgesia (TEA), CFT/ED: CFT with TEA

In the prospective arm, 60 adult patients classified as American Society of Anesthesiologists (ASA) I-II and scheduled for elective open oncologic colorectal surgery were considered eligible. Patients were excluded if they had extreme body weight (<55 kg or >120 kg), arrhythmias, recent coronary syndrome, heart failure, severe valvular disease, renal dysfunction (serum creatinine >2.0 mg/dL), communication barriers, or refusal to provide consent. Post-enrollment, patients were excluded if pulse-contour analysis could not be calibrated or if intraoperative arrhythmias occurred. A total of 49 patients were finally included in the analysis (Figure 1). Eligible participants were randomly assigned to one of two anesthetic protocols: the GDFT group, receiving GDFT with intravenous analgesia, and the GDFT/ED group, receiving GDFT with TEA. Randomization followed a computer-generated 1:1 sequence. Allocation was performed according to the randomization list at the time of enrollment.

In the retrospective arm, we reviewed the medical records of ASA I & II patients who underwent open colorectal surgery, applying the same eligibility criteria as in the prospective arm. Patients were included if they had complete perioperative records covering intraoperative hemodynamics, fluid management, diuresis, transfusion requirements, and both baseline and postoperative laboratory testing. Records with missing or incomplete data in any of these domains were excluded. Out of 98 cases screened, 72 met the eligibility criteria and were included in the final analysis (Figure 1).

In both prospective and retrospective arms, any intraoperative event resulting in a significant deviation from an uneventful course, such as massive blood loss or the need for substantial hemodynamic support (e.g., initiation of the massive transfusion protocol or administration of high-dose vasopressors), constituted grounds for exclusion.

Perioperative management

Perioperative management followed institutional protocols. In addition to standard intraoperative monitoring, a 20-gauge radial arterial catheter was placed and connected to a Vigileo™ monitor via the FloTrac™ pressure transducer (Edwards Lifesciences, Irvine, CA, USA) in the GDFT and GDFT/ED groups. Analgesia was administered either via intermittent epidural injections of a local anesthetic-opioid mixture or through continuous intravenous remifentanil infusion, according to patient allocation. Thoracic epidural catheters were placed before induction of general anesthesia at an interspace between T7/8 and L1/2, using a standard 18-gauge Tuohy needle. In the GDFT/ED group, ropivacaine 0.75% was administered in 3-5 ml aliquots, along with fentanyl (up to 100 mcg). In the GDFT group, continuous intravenous infusion of remifentanil at 0.5-1 mcg/kg/min served as the primary analgesic strategy.

In the retrospective cohort, patients who received remifentanil-based intravenous analgesia were assigned to the CFT group, while those who received TEA were classified under the CFT/ED group.

General anesthesia was induced with propofol, fentanyl, and rocuronium and maintained with volatile anesthetics in all study cases, both prospective and retrospective. Neuromuscular blockade was titrated to maintain a Train-of-Four (TOF) ratio of 0-1/4. Tidal volume was set at 8-10 ml/kg, with positive end-expiratory pressure (PEEP) maintained at ≤8 cmH₂O in GDFT-managed patients to ensure accurate hemodynamic monitoring.

Fluid management protocol

In the GDFT and GDFT/ED groups, fluid management was guided by stroke volume (SV), cardiac output/index (CO/CI), and stroke volume variation (SVV) as measured via FloTrac. Baseline SV was established post-induction and prior to incision. If SV fell ≥10% below baseline for two or more minutes, a 250 ml crystalloid bolus was infused over 10 minutes. Maintenance fluids were given at 2 ml/kg/hour. Ephedrine (5-10 mg) and phenylephrine (50-100 mcg) were administered if fluid boluses were insufficient. Transfusions were administered to maintain hemoglobin >80 g/L. Arterial blood gases were analyzed hourly or when blood loss exceeded 500 ml. Fluid optimization continued until discharge from the Post Anesthesia Care Unit (PACU).

In the CFT group, fluid therapy was aimed at maintaining mean arterial pressure >65 mmHg, heart rate <100 bpm, and urine output >0.5 ml/kg/h. Fluid and vasopressor administration were at the discretion of the attending anesthesiologist.

Data collection and outcomes

The following variables were recorded: demographics/somatometrics, medical history, intraoperative hemodynamics, fluid and blood product administration, diuresis, duration of surgery and anesthesia, and hypotensive episodes (defined as mean arterial pressure <60-65 mmHg and/or systolic arterial pressure <100 mmHg). Although vasopressors were administered as clinically indicated, cumulative vasopressor dose and duration were not predefined outcomes and were not systematically recorded in a manner allowing for quantitative analysis. In the GDFT groups, proBNP levels were measured preoperatively and 24 hours postoperatively to assess fluid balance. proBNP measurements were not available for the retrospective CFT cohorts.

Postoperative adverse events (AEs) were monitored for 10 days or until hospital discharge. AEs included predefined surgical complications (bleeding, anastomotic leakage, surgical site infections, ileus defined as absence of bowel movement or flatus by postoperative day three to five and/or need for nasogastric tube reinsertion), common pulmonary complications, major adverse cardiac events (stroke, myocardial infarction, or cardiac arrest), renal injury, reoperation, and unplanned ICU admission. All-cause in-hospital mortality was also recorded.

Patients were considered eligible for hospital discharge when they demonstrated hemodynamic stability, absence of active infection, proper wound healing, and a return to baseline mobility. These standardized ERAS criteria were applied consistently across groups, regardless of administrative delays.

Statistical analysis

The Shapiro-Wilk test was used to assess normality for continuous variables. Quantitative data are presented as means and standard deviation (SD) or medians with interquartile range (IQR), depending on distribution. Qualitative variables are reported as absolute and relative frequencies. Comparisons between continuous variables were made using the t-test or Mann-Whitney U test, as appropriate. One-way ANOVA or the Kruskal-Wallis test was used for comparisons involving more than two groups. Dunn’s test was applied for post-hoc pairwise comparisons when the Kruskal-Wallis test indicated statistical significance, with the Sidak correction applied to adjust for multiple comparisons. Fisher’s exact test was used for categorical variables. All tests were two-tailed, with p < 0.05 considered statistically significant. Analyses were performed using Stata™ (version 13.0 MP; StataCorp, College Station, TX, USA).

## Results

Results are presented for all study groups, including those managed with GDFT and those with CFT, with or without TEA. The groups were comparable in terms of demographics and somatometrics (Table 1). Specifically, the study cohort included 121 patients undergoing elective open colorectal surgery within an ERAS protocol. Of these, 49 patients were prospectively enrolled and randomized into two groups (GDFT and GDFT/ED), while 72 patient records were retrospectively reviewed (CFT and CFT/ED) (Figure 1). The gender distribution across groups was balanced (approximately 49.6% males - 50.4% females), and the median age was 72 years (64 - 78) across the groups.

**Table 1:** Baseline cohort description. Values are mean(SD), median [IQR] or n (%), accordingly. BMI: Body Mass Index, ASA: American Society of Anesthesiologists physical status classification, LAR: low anterior colon resection, proBNP: pro-B-type natriuretic peptide, GDFT: goal-directed fluid therapy, CFT: conventional fluid therapy, GDFT/ED: GDFT with thoracic epidural analgesia (TEA), CFT/ED: CFT with TEA

	CFT (n=31)	CFT/ED (n=41)	GDFT (n=24)	GDFT/ED (n=25)	P value
Demographics & somatometrics					
Gender (m/f)	17 (54.84%)/ 14 (45.16%)	20 (48.78%)/ 21 (51.22%)	12 (50%)/ 12 (50%)	11 (44%)/ 14 (56%)	0.88
Age(years)	75 [67 – 79]	72 [64 – 78]	70 [63.5 – 79.5]	71 [62 – 74]	0.79
Weight (Kg)	74.61(15.74)	75.9(12.51)	73.4(16.35)	75.88(16.41)	0.93
Height (cm)	166.64(9.58)	166.12(7.71)	166.79(9.16)	167.08(8.97)	0.98
BMI (Kg/m^2^)	26.68(4.34)	27.59(4.53)	26.22(4.38)	26.65(4.58)	0.64
ASA (I/ II) (n, %)	9 (29.03%)/ 22 (70.97%)	20 (48.78%)/ 21 (51.22%)	6 (25%)/ 18 (75%)	5 (20%)/ 20 (80%)	0.07
Type of surgery					
Total/subtotal colectomy (n, %)	4 (12.9%)	3 (7.32%)	-	3 (12%)	-
Hemicolectomy (n, %)	26 (83.87%)	37 (90.24%)	11 (45.83%)	5 (20%)	
Sigmoid colectomy (n, %)	1 (3.23%)	1 (2.44%)	8 (33.33%)	10 (40%)	
LAR (n, %)	-	-	5 (20.83%)	7 (28%)	
Baseline proBNP(pg/mL)	-	-	129.15 [81.2 – 354.75]	152 [93 – 204.4]	0.56

**Figure 2:**
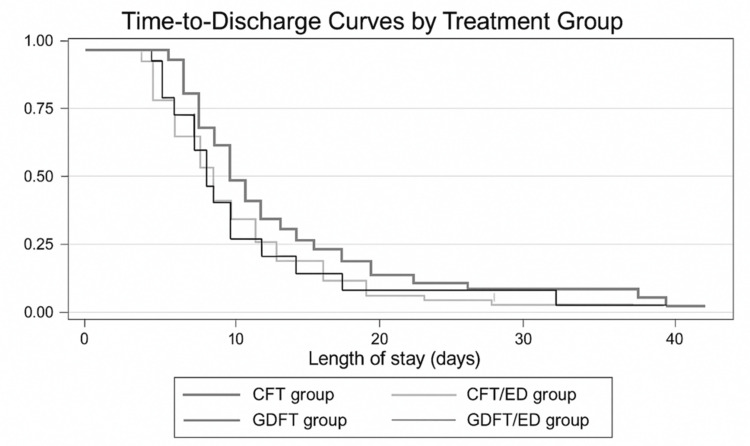
Time-to-discharge plot showing cumulative probability of discharge across study groups. Lines represent conventional fluid therapy (CFT), conventional fluid therapy with thoracic epidural analgesia (TEA) (CFT/ED), goal-directed fluid therapy (GDFT), and goal-directed fluid therapy with TEA (GDFT/ED). The x-axis represents postoperative days, and the y-axis represents the probability of remaining in hospital.

A difference in hospital length of stay across the groups was observed; however, this did not reach statistical significance (Table 2, p=0.08). Median hospital length of stay was numerically shortest in the GDFT/ED group (Table 2). These findings are further illustrated by the time-to-discharge curves depicted in Figure 2. However, pairwise comparisons revealed a statistically significant reduction in hospital stay in the GDFT/ED group compared to the CFT group (p=0.048).

**Table 2:** Perioperative data. Values are median [IQR] or n (%). Superscripts indicate significant pairwise differences (Dunn’s test, Sidak correction, p<0.05). Groups sharing the same letter do not differ significantly. Transfusion: packed red blood cells (pRBCs) and/or fresh frozen plasma (FFP), AEs: adverse events, proBNP: pro-B-type natriuretic peptide, GDFT: goal-directed fluid therapy, CFT: conventional fluid therapy, GDFT/ED: GDFT with thoracic epidural analgesia (TEA), CFT/ED: CFT with TEA.

	CFT (n=31)	CFT/ED (n=41)	GDFT (n=24)	GDFT/ED (n=25)	P value
Intraoperative data					
Fluid administration & diuresis					
Crystalloids (ml)	1625 [1300 – 1900]	1800 [1300 – 2200]	1395.5 [975 – 2135]	1500 [1150 – 2300]	0.25
Colloids (ml)	100 [0 – 500]	300 [0 – 500]	-	-	0.663
Transfusion (yes, %)	8 (25.81%)	6 (14.63%)	4 (16.67%)	2 (8%)	0.36
Total fluids (ml)	2100 [1550 – 2500]^a^	2200 [1600 – 2700]^a^	1405 [1165 – 2135]^ab^	1500 [1150 – 2500]^b^	0.006
Diuresis (ml)	300 [250 – 650]^a^	500 [300 – 600]^a^	200 [125 – 350]^b^	180 [120 – 400]^b^	<0.001
Surgical duration (min)	110 [75 – 170]	110 [80 – 160]	82.5 [62.5 – 129]	135 [75 – 170]	0.07
Postoperative data & AEs					
proBNP (pg/mL)	-	-	349.3 [267.5 – 812.5]	495 [309 – 718.3]	0.76
Length of hospital stay (d)	11 [9 – 13]^a^	9 [7 – 12]^ab^	10 [6.5 – 14.5]^ab^	9 [7 – 11]^b^	0.08
Ileus (yes, %)	2 (6.45 %)	1 (2.44%)	1 (4.17%)	-	0.65
Anastomotic leakage (yes, %)	1 (3.23%)	-	-	-	-

Intraoperative crystalloid administration was higher in the CFT groups than in the GDFT groups; however, this difference was not statistically significant (p=0.25, Table 2). Similarly, no significant differences were found in transfusion requirements between groups (Table 2). In contrast, total intraoperative fluid administration, including crystalloids, colloids, and blood products, differed significantly among the groups (p=0.006, Table 2). Patients managed with GDFT, regardless of analgesia type, received less total fluid intraoperatively. Pairwise comparisons showed a marginally significant difference in total fluid administration across all groups (p=0.064), and a statistically significant difference between the CFT and GDFT/ED groups (p=0.003). No significant difference was found between the GDFT and GDFT/ED groups (p>0.05). Diuresis was significantly higher in the CFT and CFT/ED groups compared to their respective GDFT counterparts (Table 2, p<0.001). However, there was no significant difference in diuresis between the GDFT and GDFT/ED groups (p>0.05). Regarding proBNP, measured 24 hours postoperatively in the GDFT and GDFT/ED groups, no significant differences were observed (p=0.76, Table 2).

Finally, no in-hospital mortality was recorded in all groups. Ileus occurred more frequently in the conventionally hydrated patients (CFT and CFT/ED groups), although the difference was not statistically significant (Table 2). One case of anastomotic leakage requiring reoperation was reported in the CFT group.

## Discussion

This study aimed to evaluate the impact of GDFT and TEA on hospital length of stay in low-risk patients undergoing open colorectal surgery within the ERAS protocol. Notably, our findings suggest a potential benefit from combining GDFT with TEA. Although the overall comparison across groups did not reach statistical significance, pairwise analysis revealed a significant reduction in hospital stay for the GDFT/ED group compared to the CFT group. This aligns with previous research indicating that optimized fluid management and epidural analgesia enhance recovery pathways, likely by improving pain control, minimizing postoperative complications, and preserving organ function [9,10].

Importantly, our hypothesis that TEA might predispose to overhydration through vasoplegia was not supported by the data. Although epidural-induced vasodilation was expected to increase fluid requirements, it was not associated with fluid overload, regardless of fluid management strategy. Patients in the GDFT/ED group did not receive higher intraoperative fluid volumes compared to those in the GDFT group, and postoperative proBNP levels were similar. Moreover, patients managed with GDFT, with or without ED, received significantly less total intraoperative fluid compared to those managed with CFT, reflecting the principles of individualized fluid optimization.

Postoperative AEs also favored GDFT-based management, with ileus and anastomotic leakage more frequently observed in the CFT and CFT/ED groups. Although these differences were not statistically significant, the observed trends suggest a potential clinical benefit of combining GDFT with TEA in enhancing postoperative recovery. Interestingly, Ceresoli and colleagues recently reported that adherence to restrictive intraoperative fluid management was among the strongest predictors of early recovery after colorectal surgery, while avoidance of TEA also emerged as a determinant of faster discharge [11]. These findings suggest that while fluid restriction remains universally beneficial, the role of epidurals may be more nuanced within modern ERAS frameworks. Prior studies have explored the effects of GDFT on wound infections [12], overall complications [13], ileus [14,15], renal impairment [16], long-term survival [17], and hospital stay [18-20]. However, the benefits of GDFT in low-risk patients remain inadequately studied, and the limited existing data have yielded inconsistent results. Our findings contribute to this debate by suggesting that low-risk patients may also benefit from a combined approach of GDFT and TEA.

Patient-controlled epidural analgesia (PCEA) offers superior pain relief compared to patient-controlled intravenous opioid analgesia (PCA), while also reducing opioid consumption and related side effects such as constipation and nausea/vomiting, both of which can hinder recovery after abdominal surgery. TEA has been associated with reduced ileus rates, earlier mobilization, and faster recovery [21]. However, its impact on anastomotic integrity remains debated [22]. Some evidence suggests TEA may reduce hepatic blood flow during general anesthesia [23], while other studies indicate it improves splanchnic microcirculation and protects vulnerable tissues from ischemic damage [24].

Nevertheless, recent evidence has raised questions about the incremental value of epidurals within ERAS pathways. However, when interpreted in context, these findings may reinforce the selective benefit of combining epidural techniques with individualized fluid management. The prospective multicenter study by Kato et al. in laparoscopic colectomy found no increase in complications with epidural use and even noted an earlier return of bowel function in the epidural group [25], an outcome that directly complements GDFT’s ability to preserve gastrointestinal perfusion. Moreover, although El-Boghdadly et al. concluded that regional anesthesia and analgesia showed limited additional benefits in the setting of high ERAS adherence [26], their findings suggest that epidural techniques retain their value when thoughtfully integrated into multimodal protocols. Even in low-risk colorectal patients, the combination of GDFT and epidural analgesia may provide synergistic benefits by balancing hemodynamic stability, reducing fluid overload, enhancing pain control, and ultimately accelerating recovery and shortening hospitalization.

In this study, proBNP was prospectively evaluated as a secondary marker of potential overhydration. The absence of significant differences between GDFT and GDFT/ED groups suggests that combining TEA with GDFT does not promote excessive fluid retention or cardiac strain, despite theoretical concerns that epidural-induced vasodilation might necessitate greater fluid administration. Recent studies further highlight the perioperative prognostic value of natriuretic peptides: Schmidt et al. [27] showed that preoperative N-terminal (NT)-proBNP independently predicted postoperative morbidity after non-cardiac surgery, and Zhang et al. [28] reported that elevated preoperative NT-proBNP predicted acute kidney injury (AKI) in gastrointestinal surgery under ERAS protocols, with part of the effect mediated by intraoperative infusion volume. Collectively, these data support the role of natriuretic peptides as sensitive markers of perioperative hemodynamic and fluid stress and provide context for our exploratory use of proBNP.

From a clinical standpoint, no major adverse cardiac events or in-hospital deaths were observed, supporting the safety of both GDFT and TEA in this population. Although ileus was more frequently observed in the conventionally treated groups (CFT and CFT/ED groups), the difference did not reach statistical significance. While GDFT protocols are often emphasized for high-risk patients, their utility in elective cases, especially within ERAS frameworks, remains controversial. Some studies suggest that the advantages of GDFT may be less pronounced in low-risk patients managed within a comprehensive ERAS protocol [18,19,29]. Yet, our results indicate that fluid optimization strategies may still yield meaningful improvements, particularly when combined with analgesic approaches tailored to individual patient needs.

Several limitations should be acknowledged. First, the hybrid retrospective-prospective design introduces potential sources of bias, despite the application of consistent inclusion and exclusion criteria. Second, the overall sample size was modest and no formal a priori power calculation was performed, which limits the statistical strength of our findings and may explain why certain trends did not reach statistical significance. Third, although proBNP was included as a secondary marker of fluid balance, it is an indirect measure and may not fully reflect perioperative hemodynamic status. Finally, hemodynamic guidance in the GDFT groups relied on pulse-contour analysis using the FloTrac system, the accuracy of which may be influenced by changes in vascular tone, including those induced by TEA, and should be considered when interpreting the hemodynamic data. Taken together, these limitations underscore the exploratory nature of our findings and highlight the need for larger, adequately powered, pragmatic multicenter randomized controlled trials to confirm and expand upon our observations.

## Conclusions

In conclusion, our findings suggest the use of GDFT combined with TEA as an effective strategy to accelerate recovery in low-risk patients undergoing open colorectal surgery. In this cohort, TEA was not associated with increased fluid resuscitation and was associated with shorter hospital stay in pairwise comparisons. However, our findings are limited to low-risk patients undergoing open elective colorectal surgery within an ERAS framework and should not be extrapolated to laparoscopic procedures, emergency surgery, or higher-risk surgical populations.

Overall, this retrospective-prospective cohort study suggests that integrating GDFT with epidural analgesia within ERAS pathways may enhance postoperative recovery and reduce length of stay. These exploratory results provide a rationale for future multicenter trials aimed at validating and expanding these observations.
